# Pseudopterosin and *O*-Methyltylophorinidine Suppress Cell Growth in a 3D Spheroid Co-Culture Model of Pancreatic Ductal Adenocarcinoma

**DOI:** 10.3390/bioengineering7020057

**Published:** 2020-06-14

**Authors:** Bailu Xie, Jan Hänsel, Vanessa Mundorf, Janina Betz, Irene Reimche, Mert Erkan, Ibrahim Büdeyri, Anne Gesell, Russell G. Kerr, Ni Putu Ariantari, Haiqian Yu, Peter Proksch, Nicole Teusch, Randall J. Mrsny

**Affiliations:** 1 Department of Pharmacy and Pharmacology, University of Bath, Bath BA2 7AY, UK; bailuxiepharma@outlook.com; 2 Department of Biomedical Sciences, Institute of Health Research and Education, University of Osnabrück, 49074 Osnabrück, Germany; jan.haensel@uni-osnabrueck.de (J.H.); irene.reimche@uni-osnabrueck.de (I.R.); 3 Technische Hochschule Köln, University of Technology, Arts and Sciences, 50678 Köln, Germany; V.Mundorf@gmx.de (V.M.); betzjanina@web.de (J.B.); 4 Department of Surgery, School of Medicine, Koç University, 34450 Sarıyer/İstanbul, Turkey; merkan@ku.edu.tr; 5 Research Center for Translational Medicine, Koç University, 34450 Istanbul, Turkey; Ibrahim.Buedeyri@uk-halle.de; 6 Department of Surgery, Universitätsklinikum Halle, 06120 Halle, Germany; 7 Material and Chemical Characterization Facility, University of Bath, Bath BA2 7AY, UK; Anne.Gesell@leica-microsystems.com; 8 Department of Chemistry, and Department of Biomedical Sciences, Atlantic Veterinary College, University of Prince Edward Island, Charlottetown, PE C1A 4P3, Canada; rkerr@upei.ca; 9 Institute of Pharmaceutical Biology and Biotechnology, Heinrich-Heine University Düsseldorf, 40225 Düsseldorf, Germany; Ni.Putu.Ariantari@hhu.de (N.P.A.); haiqian.yu@hhu.de (H.Y.); Peter.Proksch@hhu.de (P.P.); 10 Department of Pharmacy, Faculty of Mathematics and Natural Sciences, Udayana University, Bali 80361, Indonesia

**Keywords:** 3D co-culture, spheroid, stellate cells, PDAC, pancreatic ductal adenocarcinoma, tumor microenvironment, gemcitabine, pseudopterosin, *O*-methyltylophorinidine, p53, Ki-67

## Abstract

Current therapies for treating pancreatic ductal adenocarcinoma (PDAC) are largely ineffective, with the desmoplastic environment established within these tumors being considered a central issue. We established a 3D spheroid co-culture in vitro model using a PDAC cell line (either PANC-1 or Capan-2), combined with stellate cells freshly isolated from pancreatic tumors (PSC) or hepatic lesions (HSC), and human type I collagen to analyze the efficiency of the chemotherapeutic gemcitabine (GEM) as well as two novel drug candidates derived from natural products: pseudopterosin (PsA-D) and *O*-methyltylophorinidine (TYLO). Traditional 2D in vitro testing of these agents for cytotoxicity on PANC-1 demonstrated IC_50_ values of 4.6 (±0.47) nM, 34.02 (±1.35) µM, and 1.99 (±0.13) µM for Tylo, PsA-D, and GEM, respectively; these values were comparable for Capan-2: 5.58 (±1.74) nM, 33.94 (±1.02) µM, and 0.41 (±0.06) µM for Tylo, PsA-D, and GEM, respectively. Importantly, by assessing the extent of viable cells within 3D co-culture spheroids of PANC-1 with PSC or HSC, we could demonstrate a significant lack of efficacy for GEM, while TYLO remained active and PsA-D showed slightly reduced efficacy: GEM in PANC-1/PSC (IC_50_ = >100 µM) or PANC-1/HSC (IC_50_ = >100 µM) spheroids, TYLO in PANC-1/PSC (IC_50_ = 3.57 ± 1.30 nM) or PANC-1/HSC (IC_50_ = 6.39 ± 2.28 nM) spheroids, and to PsA-D in PANC-1/PSC (IC_50_ = 54.42 ± 12.79 µM) or PANC-1/HSC (IC_50_ = 51.75 ± 0.60 µM). Microscopic 3D rendering supported these cytotoxicity outcomes, showing little or no morphological spheroid structure change during this period of rapid cell death. Our results support the use of this 3D spheroid co-culture in vitro model having a desmoplastic microenvironment for the identification of possible novel chemotherapeutic drug candidates for PDAC, such as TYLO and PsA-D.

## 1. Introduction

Pancreatic ductal adenocarcinoma (PDAC) is the fourth leading cause of cancer death globally, having an extremely high mortality to incidence ratio [1]. Surgery, classical cytotoxic chemotherapy, radiation, or combinations of these approaches are effective only in a small number of PDAC patients, with the current five-year survival rate currently standing at ~8% [2,3]. While a number of chemotherapeutics have looked promising against PDAC cells in vitro, these agents have failed to provide durable clinical benefit; the treatment algorithm for patients in the palliative or adjuvant setting often consists of nab-paclitaxel and gemcitabine (GEM), but relapse and drug resistance are common [2]. For patients with a good performance score, in the neoadjuvant setting of borderline resectable tumors or in patients with metastatic disease Folfirinox, a combination drug regimen, became the first choice with even more toxic side effects, that extend the median survival only 2 to 4 months [4]. A lack of chemotherapeutic efficacy has been at least partially attributed to the desmoplastic and hypo-vascular microenvironment of PDAC tumors, which can limit drug access to the cancer cells. Intercellular cross-talk between PDAC cells with adjacent stromal activated stellate cells has also been shown to drive the establishment of a desmoplastic and immunosuppressive environment that not only affects tumorigenesis, angiogenesis, and metastatic spread, but is also associated with an increased cellular metabolism and efflux of chemotherapeutics [5]. On the other hand, in a Phase 1b/2 clinical trial (NCT01130142), the nonselective elimination of tumor stroma by sonic hedgehog inhibitor (IPI-926) has led to the rapid dissemination of cancer cells, causing accelerated patient death [6]. The elucidation of this unexpected result showed that the fibrotic stroma also serves as a barrier to restrain the cancer [7]. Therefore, more effective chemotherapeutics are needed that can effectively function in the microenvironment of the fibrotic stroma that creates not only challenges to drug delivery but also disadvantages to its rapid disorganization. At present, chemotherapeutics to improve the unmet medical need of effectively targeting the unique PDAC microenvironment are unlikely to be readily identified using 2D high-throughput in vitro screens with cancer cells grown on plastic substrates, urging the use of 3D cell-based models including elements of the fibrotic stroma microenvironment and reflecting the 3D tumor architecture [8,9].

Stellate cells have been identified as one of the critical elements within the PDAC tumor microenvironment, being a key cell responsible for the production of the collagenous stroma and playing an integral role of the desmoplastic stromal reaction that represents a central element of PDAC pathobiology [10,11]. Pancreatic stellate cells (PSCs) play a key role in the production of the collagenous stroma in PDAC tumors in the pancreas, interacting with cancer cells as well as several other stromal cell types (endothelial cells, immune cells and possibly neuronal cells) to promote tumor progression [10]. Hepatic stellate cells (HSCs) facilitate PDAC survival and growth following metastasis from the pancreas to the liver [12]. Interestingly, compared to primary tumors in the pancreas, a greater fraction of cancer cells are detected in tumors at metastatic sites in the liver of patients with PDAC [13], suggesting some possible differences in the organization and properties of primary pancreatic and metastatic hepatic PDAC sites that might be attributed to differences in the actions of PSCs and HSCs. We have recreated some aspects of the early phase (pre-vascularization) events of PDAC by establishing 3D spheroid cultures composed of PDAC cells and either PSCs or HSCs in the presence of the stromal element human type I collagen [14]. A main objective of our study was to evaluate the applicability as an in vitro screening tool to identify potential new drug candidates that address or overcome the fibrotic tumor microenvironment. Importantly, our current model does not include the full complement of cell types present in mature PDAC tumors, such as tumor-associated immune cells, but instead focuses on what could be considered as some of the critical early-stage events of PDAC initiation and metastasis.

Natural products isolated from a variety of sources have been shown to have clinical benefit in treating various cancers, including Vinca alkaloids, taxanes, and camptothecins. [15]. Previous screening efforts have revealed various and diverse natural product classes with strong anti-inflammatory activities in cancer cells: pseudopterosin (PsA-D) [16] and O-methyltylophorinidine (TYLO) represent two such agents, having complex structures and being isolated from biological sources. PsA-D is a mixture of four structurally related marine diterpene glycosides, which have been shown to reduce the invasiveness of triple negative breast cancer cells [16] presumably by inhibiting nuclear factor kappa B (NFκB) [17]. TYLO is a phenanthroquinolizidine alkaloid isolated from the subtropical plant *Tylophora ovata*, being developed as the analogue DCB-3503 to inhibit pancreatic cancer cell growth by targeting abnormal cell-cycle signaling [18]. Multiple analogues of TYLO have been shown to delay S Phase progression in various human cancer cells lines including pancreatic cancer [19]. 

Here, we compared the pharmacological activities of gemcitabine (GEM), currently considered as a first-line PDAC treatment in all stages of the disease [20], with the natural products PsA-D and TYLO. Our results in a novel 3D co-culture spheroid model containing PDAC cells and either PSCs or HSCs provide evidence that PsA-D and TYLO could have the ability to reduce the survival of cancer cells within PDAC tumors and may be superior to the market drug gemcitabine. 

## 2. Materials and Methods

### 2.1. Isolation Procedure of O-Methyltylophorinidine

The air-dried powdered leaves of *T. indica* (850 g) were extracted with MeOH (pH was adjusted to 11 by adding NH_4_OH) and the combined extract was evaporated in vacuo. The obtained crude extract (88.5 g) was suspended in distilled water (pH was adjusted to 2 by the addition of 0.5N H_2_SO_4_) and filtered through filter paper. The water-soluble part was then extracted with EtOAc yielding the EtOAc phase and the acidic aqueous phase. The latter was basified by adding NH_4_OH to pH 11 prior to the extraction with EtOAc, resulting in the alkaloid-containing EtOAc extract (8.2 g). The EtOAc extract was then subjected to vacuum liquid chromatography (VLC) on a diol silica column, employing *n*-hexane: EtOAc: MeOH (8:1:1) as solvents to afford 13 fractions. Purification of fraction 13 by semipreparative HPLC with MeOH and 0.1% HCOOH in H_2_O as the mobile phase yielded *O*-methyltylophorinidine (20 mg).

*O*-methyltylophorinidine: a yellow amorphous, solid; [α${]}_{D}^{20}$ +44 (*c* 0.1, CDCl_3_); *m/z* 380 [M + H]^+^; ^1^H, ^13^C and 2D NMR data were in close agreement with those reported in the literature [16].

### 2.2. Preparation of PsA-D Mixture

*A. elisabethae* was collected from South Bimini Island, The Bahamas, and was dried and extracted in EtOAc/MeOH (1:1) for 48 h. The crude extract was subjected to silica gel chromatography eluting with hexanes and EtOAc to afford a mixture of PsA-D [21]. The ratio was determined to be 85:5:5:5 (PsA:B:C:D) by LC–MS analysis.

### 2.3. Cell Culture

Human pancreatic cancer cell lines Capan-2 and PANC-1 were obtained from the American Type Culture Collection (Manassas, VA, USA). Cells were maintained in a DMEM cell culture medium with high-glucose (4 g/L) (GibcoTM, Cat. # 41965-039) supplemented with 10% fetal bovine serum (FBS, GibcoTM, Cat. # 10500064), and 100 U/mL penicillin combined with 100 mg/mL streptomycin (P/S, Sigma-Aldrich Chemical Co., Munich, Germany, Cat. # P4333). Patient-derived hepatic and pancreatic stellate cells were generous gifts from Dr. Erkan at Koç University hospital, Turkey. Ethical approval was obtained from the Ethics Committee for Biomedical Sciences of KOҪ University and written informed consent was obtained from all the patients. Sterile tissues were obtained immediately after the surgical resection of pancreatic tumors and liver metastatic sites from patients diagnosed with pancreatic ductal adenocarcinoma. Human stellate cell isolation and cultivation were performed under sterile conditions for all cell types. Stellate cells were maintained in a DMEM/F12 cell culture medium containing DMEM with low-glucose (1 g/L) (GibcoTM, Cat. # 22320022) and Ham’s F-12 Nutrient Mix (GibcoTM, Cat. # 21765029) at 1:1 (volume/volume) supplemented with 20% FBS and P/S as described [22]. All the cells were routinely cultivated in a humidified incubator with 5 % CO_2_ at 37 °C.

### 2.4. Preparation of PolyHEMA Low-Attachment Plates

PolyHEMA low-attachment plates were prepared as described previously [23]. A 120 mg/mL stock solution of poly-HEMA (Sigma-Aldrich Chemical Co., Cat. # P3932) was incubated while stirring with a magnetic bar at room temperature (15–20 °C) overnight. To make a working solution of poly-HEMA, 1 mL of poly-HEMA stock solution was pipetted into 23 mL of 95% ethanol to obtain a final concentration of 5 mg/mL. The fresh working solution was prepared every time new plates were made. Then, 50–60 μL of poly-HEMA working solution was pipetted into each well of a 96-well U-bottomed plate (Nunc^TM^, Cat. # 163320). The ethanol was evaporated at 37 °C for 72–96 h under humid-free conditions. Before use, the plates were sterilized in the hood with the lids off using UV light for 40–60 min. Sterilized plates were sealed with Parafilm and stored at room temperature.

### 2.5. Establishment of 3D Co-Culture PDAC Models

Stellate cells were isolated and cultivated as published previously [24], with ethics committee approval for the collection of PSC and HSC obtained at Koc University School of Medicine (2015.167.IRB2.064) under the International Ethical Guidelines for Biomedical Research Involving Human Subjects (CIOMS) guidelines. Pancreatic cancer cells obtained from the American Type Culture Collection (ATCC) were grown to reach 60–90% confluence using the ATCC-suggested media conditions. Cells were trypsinized and lifted using 0.25% trypsin with 0.02% EDTA at 37 °C, with the process being halted by the medium composed of DMEM/F12 + 10–20% FBS + P/S. Cell count in the collected cell preparations was determined using a glass hemocytometer or a LUNA-II™ Cell Counter (Logos Biosystems, Anyang-si, South Korea). Mixtures of stellate cells and cancer cells were prepared at a 2:1 ratio to produce a final cell suspension of 1000 stellate cells and 500 cancer cells per 50 µL (20,000 cells/mL stellate cells and 10,000 cells/mL cancer cells), in each well of a 96-well plate. Tubes with the stellate cells and cancer cells mixture were centrifuged at 125 g for 4–5 min and the supernatant was aspirated to discard trypsin. Cell pellets in the tubes were resuspended in an ice-cold medium (DMEM/F12 + 10% FBS + P/S) with the volume matching the cell suspension of 20,000 cells/mL stellate cells and 10,000 cells/mL cancer cells. Collagen I was added to reach the final concentration of 0.1 mg/mL and mixed well, keeping the tubes on ice packs as long as possible. Subsequently, 50 µL cell–collagen mixture was pipetted into each well of the 96-well polyHEMA plates, Corning^®^ 96-well Black/Clear Round Bottom Ultra-Low Attachment Spheroid Microplates (Corning, Cat.# 4515), or CELLSTAR^®^ PS Microplates (Greiner Bio-One, Cat.#650970) at room temperature. Finally, 50 µL fresh medium DMEM/F12 + 10% FBS + P/S was added the next day. The outside of the plates can be gently tapped using fingers at the small area of wells to facilitate the spheroids that stay alongside the walls of the wells to drop to the bottom of the wells.

### 2.6. 2D Cell Proliferation Assay

Capan-2 and PANC-1 cancer cells were grown to reach 50–90% confluence. The cells were trypsinized and lifted using 0.25% trypsin with 0.02% EDTA at 37 °C, with the process being halted by a medium composed of DMEM + 10% FBS + 1% Pen/Strep. The cell count in the collected cell preparations was determined using a glass hemocytometer or a LUNA-II™ Cell Counter (Logos Biosystems, Anyang-si, Korea). Cancer cells were seeded in black Advanced TC™- 96 Well Cell Culture Microplate (Greiner Bio-One, Frickenhausen, Germany, Cat.# 655986) or in a clear TC™-96 Well Cell Culture Microplate (Sarstedt, Nümbrecht, Germany, Cat.# 83.3924) at the density of 10,000 cells per well for the Capan-2 cells, and 5000 cells per well for the PANC-1 cells. After 24 h initial incubation, 10 mM gemcitabine (GEM) hydrochloride (Santa Cruz Biotechnology, Dallas, TX, USA, Cat. # sc-204763), 10 mM Pseudopterosin A-D (PsA-D) from Prof. Dr. Russell Kerr, Veterinary College, University of Prince Edward Island, Canada, and 10 mM *O*-methyltylophorinidine (TYLO) from Prof. Dr. Proksch, Institute of Pharmacological Biology and Biotechnology, Heinrich-Heine-University Düsseldorf, were all dissolved in dimethyl sulfoxide (DMSO, Sigma, Munich, Germany, Cat.# D2650) and added to reach the final concentrations of 100 µM GEM, from 100 to 1.5 µM (seven 1:2 dilution steps) PsA-D and from 1 µM to 1.4 nM (seven 1:3 dilution steps) TYLO, respectively, in a total volume of 100 µL and triplicates for each measuring point. After a 48–72 h treatment, cell viability was determined by applying the CyQUANT™ NF Cell Proliferation Assay (ThermoFisher, Eugene, USA, Cat.# C35006) or by using the CellTiter-Glo^®^ Luminescent Cell Viability Assay (Promega, Fichtburg, MA, USA, Cat.# G7570) as indicated in the manufacturer’s protocol. Fluorescence intensity was measured by FLUOstar Omega spectrometer (BMG, Ortenberg, Germany), and the luminescence intensity by TECAN Spark (TECAN, Männedorf, Switzerland)**.** Each experiment was repeated at least three times.

### 2.7. 3D Cell Viability Assay after Treatments of Anti-Cancer Agents on the 3D Co-Culture PDAC Models in Collagen Matrix

The three-dimensional co-culture was conducted as described above. The final concentrations of 100 µM GEM (positive control), from 100 to 1.5 µM (seven 1:2 dilution steps) PsA-D and from 1 µM to 1.4 nM (seven 1:3 dilution steps) TYLO, respectively, were used. The concentration of 100 µM GEM was chosen according to the IC_50_ previously reported [25]. The concentrations of 70 µM PsA-D and 0.1 µM TYLO were chosen based on the previous IC_50_ results (lab Nicole Teusch, unpublished results). Both 0.7% and 1% DMSO, depending upon the conditions used to examine various test agents, were used as solvent controls with 10% FBS being added to the untreated controls. After 72 h treatments, the cell viability was determined by CellTiter-Glo^®^ 3D Cell Viability Assay (Promega, Fichtburg, MA, USA, Cat.# G9683/G9681) according to manufacturer’s specifications. Luminescence was measured by CLARIOstar (BMG LABTECH, Ortenberg, Germany) or Tecan SPARK (TECAN, Männedorf, Switzerland). Of note, when the spheroids were grown and treated in poly-HEMA plates, the whole content of each well was transferred to white Advanced TC™- 96 Well Cell Culture Microplate (Greiner Bio-One, Cat.# 655983) for the measurement of luminescence. Spheroids grown in CELLSTAR^®^ PS Microplates (Greiner Bio-One, Cat.# 650970) were transferred to black 96 Well Microplates (Greiner Bio-One, Cat.# 655090) in order to measure the luminescent signals as well.

### 2.8. Phase-Contrast Imaging

Phase-contrast images of 3D co-culture PDAC models were acquired by Leica DMI4000 B (Leica Microsystems, Wetzlar, Germany); (50× magnification) manually at the indicated time points. At least three wells of each spheroid group were acquired in each experiment except one well of the spheroid groups untreated with agents and treated with GEM in one independent experiment. 

### 2.9. Immunofluorescence on Paraffin-Embedded Sections

At least 10 spheroids grown for 7 days were transferred to 1.5 mL microcentrifuge tubes. After the aspiration of cell culture medium as much as possible, spheroids were fixed in >500 µL 4% (*w*/*v* in PBS) paraformaldehyde (PFA, Sigma-Aldrich Chemical Co., Cat. # P6148) at room temperature for > 1 h. After the removal of PFA, the spheroids were rinsed 4 times in 3 mg/mL polyvinylpyrrolidone (PVP, Sigma, Cat. # P0930) in PBS (PBS/PVP) and then stored at 4 °C for no more than 2 weeks until processing. After removing the PBS/PVP as much as possible, 100 µL 2% (*w*/*v* in dH_2_O) warm liquid agarose (Fisher, Cat. # BP1356-100) was added to each tube and the liquid agarose with spheroids was transferred to the lid of each corresponding 1.5 mL tube to solidify overnight at 4 °C. The agarose blocks were transferred to histology cassettes and incubated in 50% ethanol for 1 h and then 70% ethanol until processing. After tissue processing, agarose blocks were embedded in paraffin, which were cut to produce 5 µm sections using a microtome (Leica Jung 2035). Paraffin-embedded sections, placed on slides, were incubated in two washes of Histo-Clear II (National diagnostics, Cat.# HS-202) for 5 min each, one wash of 100%, 90%, 80%, and 70% ethanol for 5 min each, and three washes of PBS for 5 min each. Antigen retrieval was facilitated by boiling slides in 100 mM pH 6.0 sodium citrate buffer for 30 s. After washing thrice in PBS/PVP for 5 min, the paraffin-embedded sections on slides were permeabilized with 0.25% Triton™ X-100 in PBS/PVP for 30–45 min, incubated with 100–400 µL blocking solution (0.1% BSA and 0.01% Tween 20 in PBS) for 15–30 min at room temperature, and incubated with 100–400 µL of a primary antibody (1:100) overnight at 4 °C overnight. After washing thrice in PBS for 5 min, the paraffin-embedded sections were incubated with secondary antibodies solution (1:200 in blocking solution) and 4′,6-diamidino-2-phenylindole dihydrochloride (DAPI, Sigma, Cat. #D9542) in a humidified chamber for 1.5 h at room temperature. After secondary antibodies’ incubation, the slides were washed thrice in a blocking solution for 5 min before dehydration using a 70%, 80%, 90%, 100% series of ethanol in MilliQ water, followed by coverslip mounting using Fluoroshield Mounting Medium (Abcam, Cat. #ab104135).

### 2.10. Immunofluorescence on Entire Spheroids

Three-dimensional co-culture PDAC spheroids were either collected in 1.5 mL microcentrifuge tubes on day 4 or on day 7 without or with treatments of 100 µM gemcitabine, 70 µM PsA-D and 0.1 µM *O*-methyltylophorindine. Then, 0.7% DMSO was used as the solvent control and a fresh medium with 10% FBS was used as the untreated control. Immunofluorescence staining of the cells within the 3D spheroids was performed as described [23] with slight modifications. After the aspiration of the cell culture medium as much as possible, 3–10 spheroids were fixed in 4% PFA in PBS using Eppendorf tubes for 0.5–2 h at room temperature. After fixing, the spheroids were rinsed 4 times in 3 mg/mL PBS/PVP and then stored at 4 °C for no more than 2 weeks before processing. Spheroids were permeabilized in 0.25% Triton™ X-100 (Sigma, Cat. # X100) in PBS/PVP for 0.5–1 h and placed in a blocking solution (0.1% BSA and 0.01% Tween 20 in PBS) for 0.5–1 h. Spheroids were incubated in primary antibodies solution (1:100 in blocking solution) or the mixture of goat IgG, rabbit IgG and mouse IgG for 19–24 h with gentle shaking in the 4 °C, rinsed thrice for 15 min at room temperature and incubated in a secondary antibody solution (1:200 in blocking solution) and 4′,6-diamidino-2-phenylindole dihydrochloride (DAPI, Sigma, Cat. #D9542) with gentle shaking at 4 °C overnight with protection from the light. After rinsing, the samples were dehydrated through 30%, 50%, 70%, 80%, 90%, 100% series of ethanol in MilliQ water. After the aspiration of the residual ethanol, the spheroids were incubated in benzyl alcohol (ThermoFisher, Alfa Aesar, Kandel, Germany Cat. #L03292) and benzyl benzoate (ThermoFisher, Alfa Aesar, Kandel, Germany, Cat. #L03258) at 1:2 (*v*/*v*, BABB) for at least 1 hr at room temperature, before being transferred to the wells of an ibidi µ-Plate 96 well black (ibidi, Cat. #89626) with as little BABB as possible to prevent the movement of spheroids in BABB so that the spheroids stayed stationary in the wells. Plates were stored at 4 °C until analysis.

### 2.11. Confocal Laser Scanning Microscopy (CLSM)

The images were acquired on a Zeiss 880 confocal laser scanning microscope with Airyscan (Carl Zeiss, Jena, Germany) using a Plan-Apochromat 10×/0.45 NA objective lens (2 mm working distance) below 20 °C. Fluorophores were excited simultaneously using at least three different laser wavelengths as specified below, a 488/561/633 main beam splitter and a −405 main beam splitter InVis with the non-descanned detector to acquire the differential interference contrast (DIC) images. The lasers and filter settings were as follows: 405 nm (Argon laser) excitation and 410–497 nm filters for DAPI; 488 nm (Argon laser) excitation and 500–553 nm filters for Alexa Fluor 488; 561 nm (Argon laser) and 562–624 nm filters for Alexa Fluor 546; 633 nm (HeNe laser) and 624–735 nm filters for Alexa Fluor 647. The multitracking mode separating each channel into one track individually were used.

### 2.12. Image Analysis

Histogram adjustment, orthogonal view, 3D rendering, and image exporting were performed using Zen 2.3 (blue edition). The channels collecting the fluorescence from the DAPI staining of the nuclei were pseudo-colored blue. The channels collecting the fluorescence from the goat anti-p53 antibodies with the donkey anti-goat secondary antibody with Alexa Fluor 488, and the mouse anti-p53 antibodies with the donkey anti-mouse secondary antibody with Alexa Fluor 647, were pseudo-colored green. The channel collecting the fluorescence from the rabbit anti-αSMA antibodies or the rabbit anti-Ki67 antibodies with donkey anti-rabbit secondary antibody with Alexa Fluor 546 were pseudo-colored red. The channels collecting the fluorescence from IgGs were pseudo-colored according the species of the antibodies used. After positioning the orthogonal view and the 3D rendering rotation, images were collected.

### 2.13. Antibodies

Commercial sources of antibodies and the dilutions used in these studies are shown in Table 1.

### 2.14. Statistical Analysis

Obtained data represent at least three independent biological experiments (n = 3). Error bars show ± SEM of the means of triplicate values. Data analysis, evaluation, and illustration was conducted applying Excel 2019 (Microsoft Office) or were generated with Graphpad Prism v. 8.0 (Graphpad Software, San Diego, CA, USA).

## 3. Results

### 3.1. Establishment and Properties of 3D PDAC-Stellate Cell Co-Culture Models

**Figure 1:**
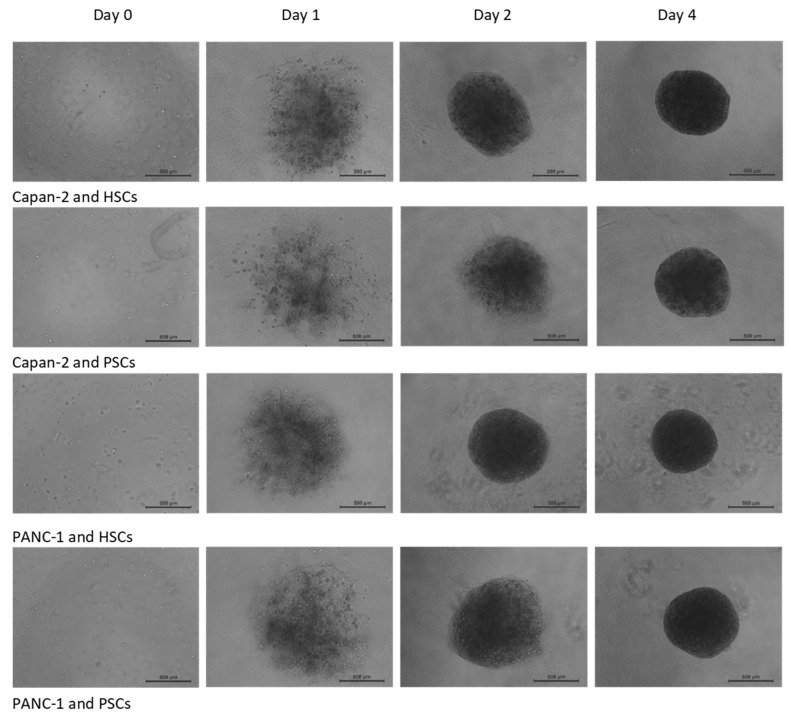
Time-course of 3D spheroid formation: 500 pancreatic ductal adenocarcinoma (PDAC) cells and 1000 hepatic stellate cells (HSCs) or pancreatic stellate cells (PSCs) were mixed in 50 μL of 0.1 mg/mL human type I collagen as indicated. Phase-contrast images were acquired (50× magnification) manually at the indicated time points. Data shown were representative images from at least three independent experiments with at least three spheroids in each experiment. Scale bar: 500 μm.

The avascular phase of solid tumor metastasis, limited by oxygen and nutrient diffusion, becomes untenable beyond roughly 0.5–1 million cells or ~1–2 mm in diameter [26]. In order to recreate such an early-stage micro-tumor condition relevant for PDAC, we established an in vitro 3D co-culture model composed of an established PDAC cell line (Capan-2 or PANC-1) and isolated primary stellate cells from pancreatic tumors (PSCs) or hepatic metastatic sites (HSCs) obtained from PDAC resections [24]. To aid in the initial establishment of these 3D spheroids, a mixture of stellate to cancer cells at a 2:1 ratio, which has been used previously in PDAC modeling [27,28,29], was combined with one of the major extracellular matrix (ECM) elements in the PDAC environment: human type I collagen [30,31,32]. Specifically, ~500 cancer cells and ~1000 stellate cells were suspended in 50 μL of media containing 0.1 mg/mL human type I collagen and introduced into each well of a polyHEMA coated low-attachment 96-well plate. It should be noted that this method used much less type I collagen than has been described for other 3D PDAC models where concentrations of 1–2 mg/mL were used [27,31,32]. On day 1 after seeding, a loose aggregate could be seen in each well (Figure 1). Following 4 days of culture, 3D spheroids of ~0.5–1 mm in diameter were consistently established as monitored by phase-contrast microscopy (Figure 1). 

**Figure 2:**
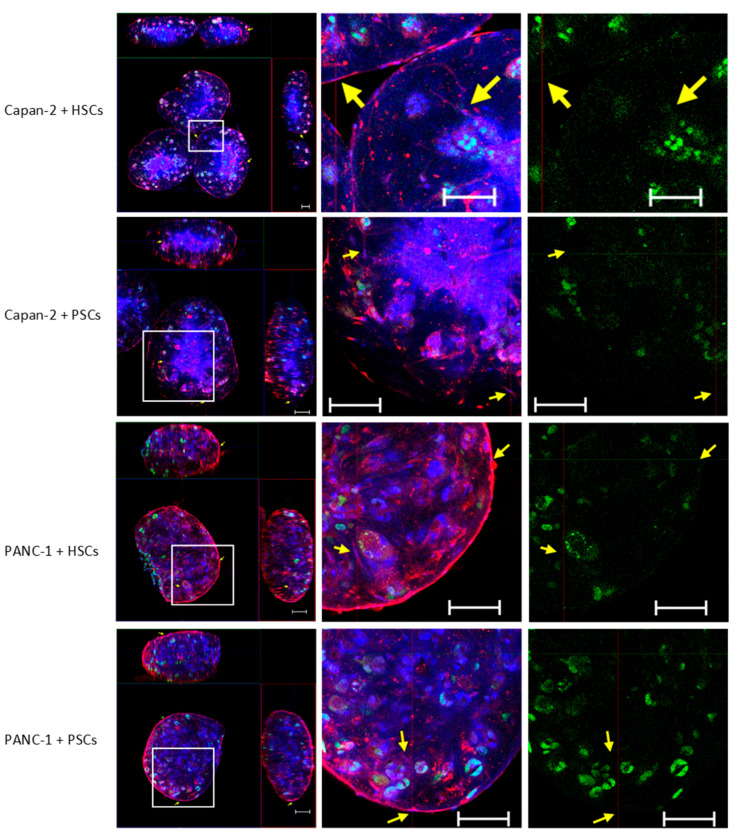
Distribution of the stellate and cancer cells in 3D spheroids using confocal laser scanning microscopy. At day 4 of culture, spheroids formed from 500 cancer cells (PANC-1 or Capan-2) and 1000 hepatic stellate cells (HSCs) or pancreatic stellate cells (PSCs) in 50 μL of 0.1 mg/mL human type I collagen were fixed and stained for α-SMA (red) and p53 (green), with DAPI (blue) being used to demonstrate the nuclei. Yellow arrows indicate stellate cells. Images are representative from at least three independent experiments with at least three spheroids in each experiment. Scale bar: 100 μm.

Alpha smooth muscle actin (α-SMA) has been widely used as a marker for activated stellate cells [33]. The tumor suppressor protein p53 is commonly overexpressed in PDACs and has a missense mutation that disrupts its normal anti-proliferative properties, giving it a gain-of-function activity that promotes metastasis [34]. Confocal laser scanning microscopy was used to determine αSMA expression patterns alongside the distribution of p53 to discriminate stellate cells from cancer cells within 3D spheroids. X and Y image projections showed comparable and relatively uniform distributions of α-SMA-positive activated stellate cells with a characteristic star-like morphology [35], and p53-positive cancer cells in these 3D spheroids (Figure 2). Cancer cells were observed in clusters disseminated throughout the 3D spheroid matrix, with stellate cells (yellow arrows) present at the spheroid surface and integrated throughout the matrix observed (Figure 2). 

No striking differences in the properties of the cancer cell numbers and the organization/distribution within the 3D spheroids formed using Capan-2 or PANC-1 cells were observed when co-cultured with either HSCs or PSCs (Figure 2). To further examine this point, confocal laser scanning microscopy Z-stack imaging and 3D rendering was used to examine these spheroids. Capan-2 or PANC-1 cells were observed to be distributed comparably in the 3D spheroids, with the stellate cells dispersed in a relatively uniform manner (Figure S1). 

To verify that a higher resolution assessment of cancer cell distribution and properties could be examined using confocal laser scanning microscopy, we compared Z-stack imaging and 3D rendering with images of spheroids obtained from ~5 μm thick sections of fixed, paraffin-embedded samples (Figure S2). The distribution of p53 and Ki67, nuclear markers that have been used to assess proliferation and define the growth properties of a cell population [36], were comparable for both physically- and optically-sectioned samples. While there was superior microscopic resolution for the physically sectioned samples, these two protocols were sufficiently comparable to suggest that optical imaging could provide accurate assessments of cancer cell properties within these 3D spheroids. 

### 3.2. Cytotoxicity of Gemcitabine, Pseudopterosin A-D, and O-Methyltylophorinidine in 2D versus 3D PDAC Models

**Figure 3:**
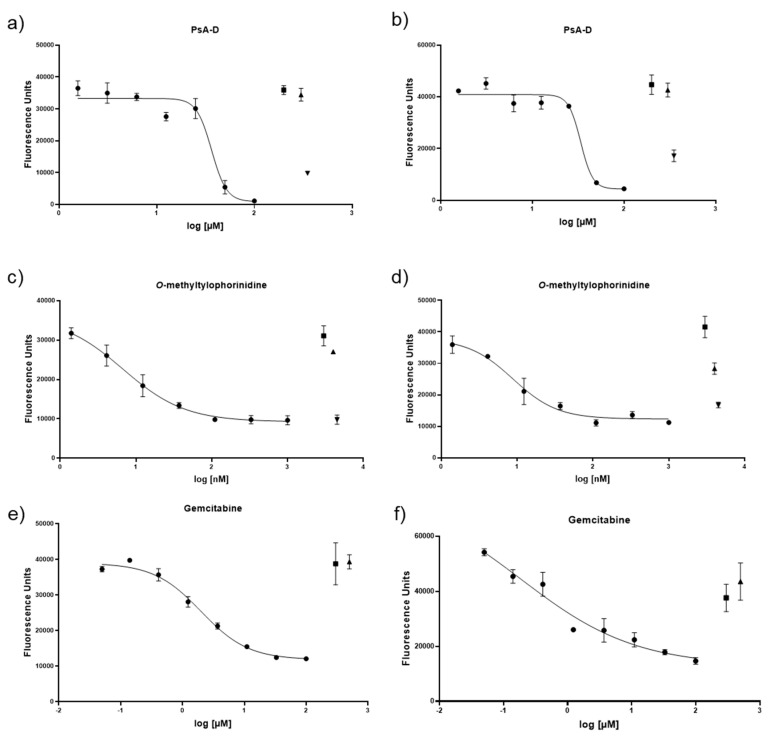
Comparison of the cell viability response curves for the two pancreatic cancer cell lines exposed to pseudopterosin A-D (PsA-D), *O*-methyltylophorinidine (TYLO), or gemcitabine (GEM) in a 2D format: (**a**) PsA-D on Panc-1; (**b**) PsA-D on Capan-2; (**c**) TYLO on Panc-1; (**d**) TYLO on Capan-2; (**e**) GEM on Panc-1; and (**f**) GEM on Capan-2. Culture wells were seeded with 10,000 cells per ml for the Capan-2 or 5000 cells per ml for the PANC-1 cells, which were treated with a dose range of test agent for 72 h, when the cell viability was assessed using the CyQUANT™ NF Cell Proliferation Assay. In all cases, DMSO (1.0%) was present to ensure agent solubility and was present in the control tests as an untreated control (▲: DMSO 1 %; █: cell culture media), while GEM served as a positive control (▼: Gemcitabine (100 µM)). Data points represent at least n = 3 means ± SEM.

We examined the efficacy of pseudopterosin A-D (PsA-D), *O*-methyltylophorinidine (TYLO), or gemcitabine (GEM) at 48 h in the PDAC cells Capan-2 or PANC-1, when grown in a standard 2D format (Figure 3, Table 2). IC_50_ values determined in the 2D model demonstrated a TYLO > GEM > PsA-D hierarchy of efficacy. There was limited information for the cytotoxic actions of these agents on these cell lines, but for comparison, the IC_50_ for the GEM on PANC-1 cell survival was previously determined in 2D cell culture with 9.5 μM [37]. The course of the dose–response curves for these three drugs (candidates) in the 2D model were somewhat distinctive; PsA-D demonstrated a relatively steep sigmoid-shaped dose–response curves, while GEM and TYLO demonstrated more gradual sigmoid-shaped curves (Figure 3).

**Figure 4:**
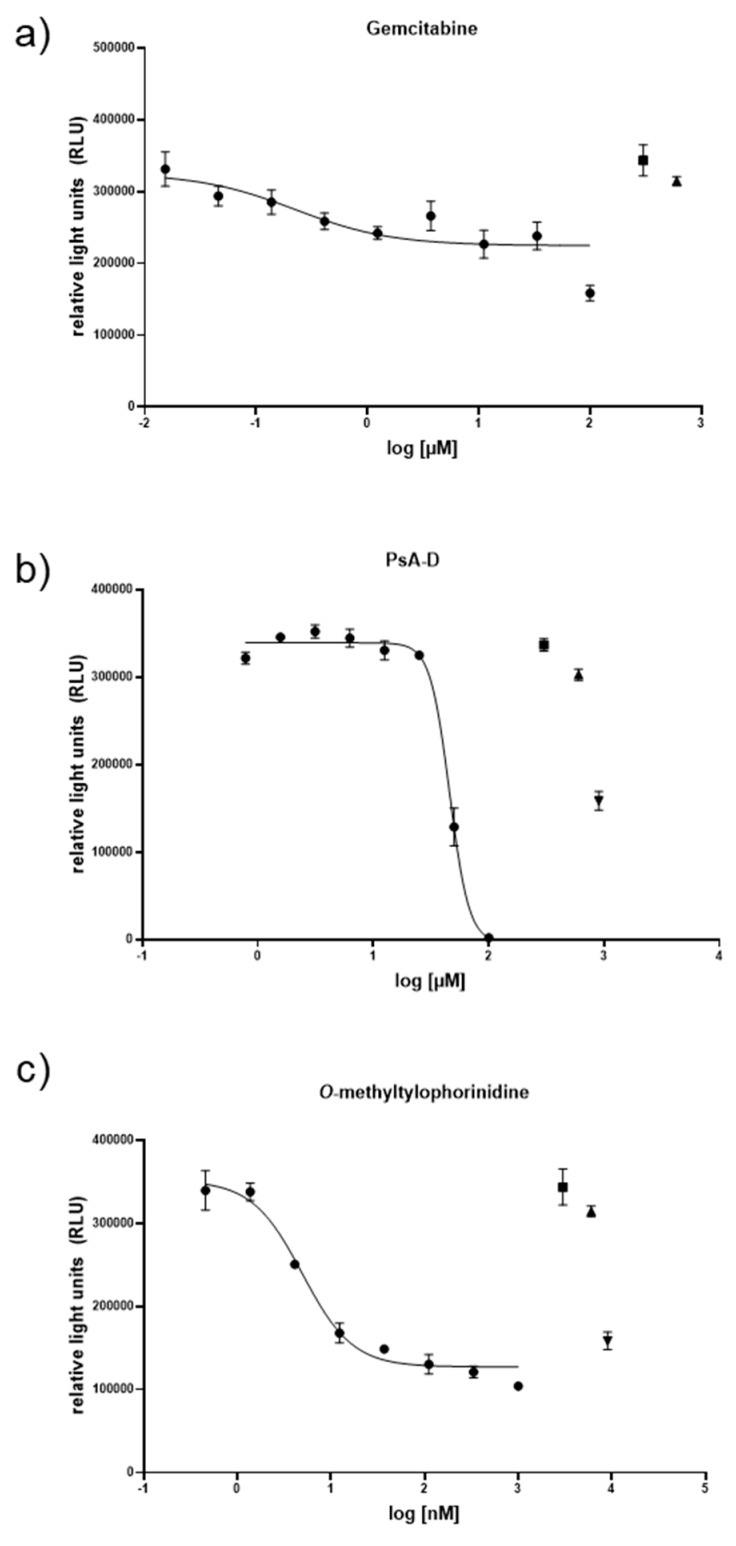
Comparison of the cell viability response curves for the Panc-1 + pancreatic stellate cell (PSC) spheroids exposed to (**a**) gemcitabine, (**b**) pseudopterosin A-D (PsA-D), and (**c**) *O*-methyltylophorinidine. Co-cultured spheroids were seeded with 50 µL 10,000 Panc-1 cells per ml plus 20,000 PSC cells per mL, grown for 72 h and treated with a serial dilution series of test agents for another 72 h, when the cell viability was assessed using the CellTiter-Glo^®^ 3D Cell Viability Assay (Promega). In all cases, DMSO (0.7%) was present to ensure agent solubility and thus, served as an untreated control (▲: DMSO 0.7 %, █: cell culture media), while the GEM served as a positive control (▼: Gemcitabine (100 µM)). Data points represent at least n = 3 means ± SEM.

As the responses for Capan-2 and PANC-1 in 2D were comparable for PsA-D, TYLO, or GEM, we focused only on the latter PDAC cell line for an initial comparison to the 3D co-culture model format (Figure 4). PANC-1 was demonstrated to grow faster than Capan-2 in traditional 2D cultures [38], presumably providing a more aggressive test system for the 3D format of spheroids prepared with human type I collagen and either PSCs or HSCs. Notably, in our 3D co-culture format, TYLO conserved its striking efficacy in the low nanomolar range (Figure 4, Table 3), while PsA-D was less effective than in the 2D model with a right based shift of ~2-fold for PsA-D (Figure 4, Table 3). Most remarkably, GEM lost efficacy by around 50-fold in the PANC-1/PCS co-culture spheroids and by around 240-fold in the PANC-1/HCS spheroids relative to its activity observed in the 2D model. Overall, these results were consistent with the hypothesis that cancer cells cultured in the presence of stellate cells, which are key players in creating the highly fibrotic tumor microenvironment, can become more resistant to currently approved chemotherapeutics when compared to cancer cells alone [39]. Of the agents tested, this difference was most striking for GEM and was consistent with previous findings [40].

### 3.3. Alteration of p53 and Ki67 in 3D Co-Culture PDAC Models

**Figure 5:**
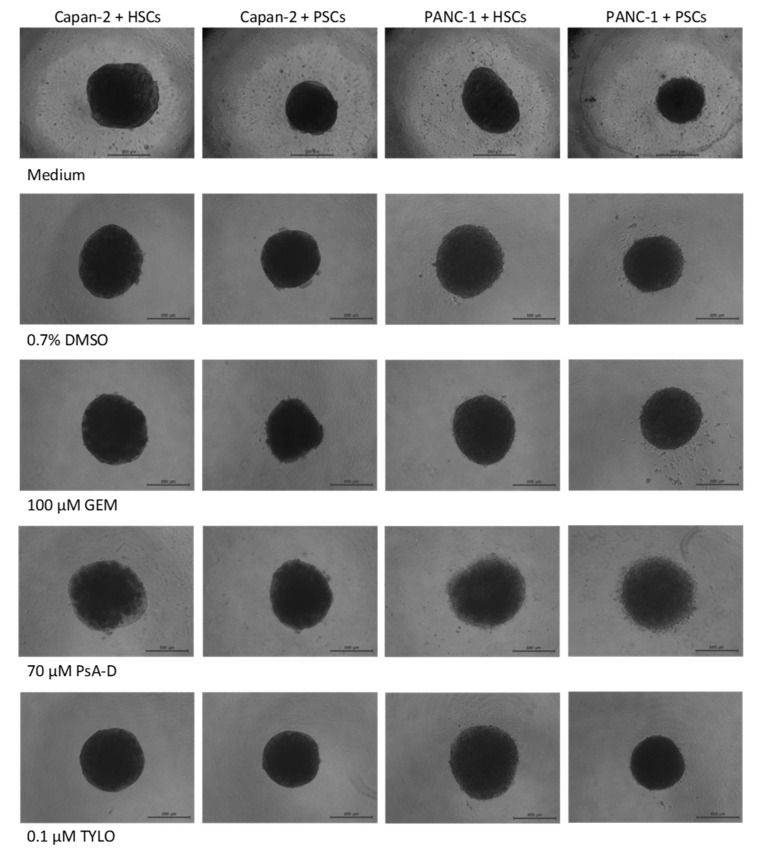
Phase-contrast images of the 3D spheroids prepared with 500 Capan-2 or PANC-1 cancer cells and 1000 hepatic stellate cells (HSCs) or pancreatic stellate cells (PSCs) in 50 μL of 0.1 mg/mL human type I collagen. After 4 days in culture, the spheroids were treated with medium, 100 μM gemcitabine (GEM), 70 μM pseudopterosin A-D (PsA-D), 0.1 μM *O*-methyltylophorinidine (TYLO), and 0.7% DMSO as a solvent control. After 3 additional days, the spheroid size and morphology were examined. Images are representative from at least three independent experiments with at least three spheroids in each experiment. Scale bar: 500 μm.

Based upon the cytotoxicity results described above for the 3D model, we considered the possibility that the increased variability compared to the 2D outcomes could be due to differences in the susceptibility of cells within the spheroid matrix. To this end, 70 μM PsA-D, or 0.1 μM TYLO, or 100 μM GEM were added to the 3D co-culture spheroids prepared from cancer cells and stellate cells with type I collagen after 4 days of growth in vitro. After 3 additional days of agent exposure, phase-contrast images showed no striking differences in the general morphology between the control spheroids compared to any of the treatments, other than the PsA-D treatment group where the surface of these spheroids appeared somewhat less organized (Figure 5). These results suggested that the matrix format of the 3D spheroids was not dramatically impacted by the actions of the drug candidates and that the assessment of the cell status within the spheroid matrix will be important to examine their pharmacological impact.

**Figure 6:**
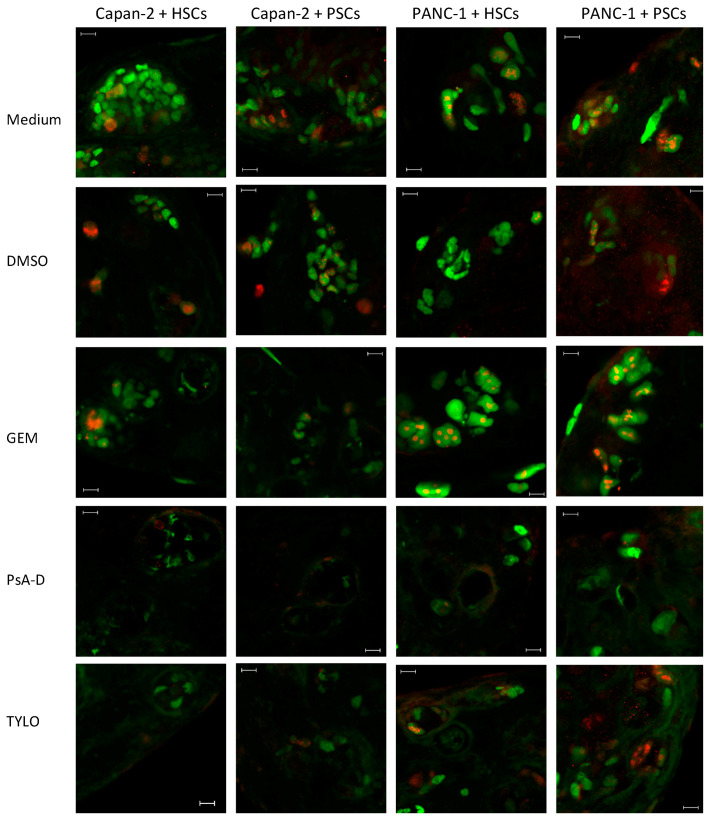
Optical sections demonstrating THE Ki-67 (red) and p53 (green) expression using Airyscan imaging near the edge of intact spheroids. Spheroids prepared from 500 cancer cells and 1000 hepatic stellate cells (HSCs) or pancreatic stellate cells (PSCs) in 50 μL of 0.1 mg/mL human type I collagen were grown for 4 days and then treated with the medium, 100 μM gemcitabine (GEM), 70 μM pseudopterosin A-D (PsA-D), 0.1 μM *O*-methyltylophorinidine (TYLO), and 0.7% DMSO as a solvent control. Scale bar: 10 µm.

To examine the cellular responses within the matrix of our 3D model, the spheroids were stained for two proteins to specifically define the cell status: the tumor suppressor protein p53, which is extremely frequently mutated in PDAC tumors [41] and the proliferation marker Ki-67 [42]. Airyscan images of the whole spheroids were used to examine the 3D distribution of these two proteins. In the co-cultures, both p53 and Ki-67, were readily detectable in the PDAC cells. Notably, and as expected, p53 showed a nuclear distribution, while Ki-67 was more restricted to some regions inside the nucleus [43]. Importantly, there was variability of PDAC and stellate cell distribution within each 3D spheroid, making the acquisition of the representative images to describe the impact of GEM, PsA-D, and TYLO in various combinations of the PDAC cells Capan-2 and PANC-1 with either HSCs or PSCs somewhat challenging (Figure 6). While we attempted to obtain representative images that reflected the changes in the properties of p53 and Ki-67 induced by GEM, PsA-D, and TYLO, the cells surviving these treatments were dramatically reduced in number and were altered in their appearance in most cases, making it even more difficult to compare the overall extent of pharmacological actions in these various 3D spheroid composition scenarios. In general, we observed that spheroids containing Capan-2 cells showed greater losses in the total cell number and altered their appearance compared to those containing PANC-1 cells and that PsA-D and TYLO were more effective than GEM under the conditions tested for both, Capan-2 and PANC-1 spheroids (Figure 6).

Having verified that the overall nature of the 3D matrix was not dramatically altered but that cellular responses had been achieved within the spheroids treated, particularly with PsA-D and TYLO, we explored a 3D rendering method to allow for the better characterization of drug actions in this model. Notably, spheroids treated with 0.7% DMSO, the concentration used for the solubilization of PsA-D, TYLO, and GEM treatments, showed no effect on cell distribution within these 3D matrices (data not shown).

**Figure 7:**
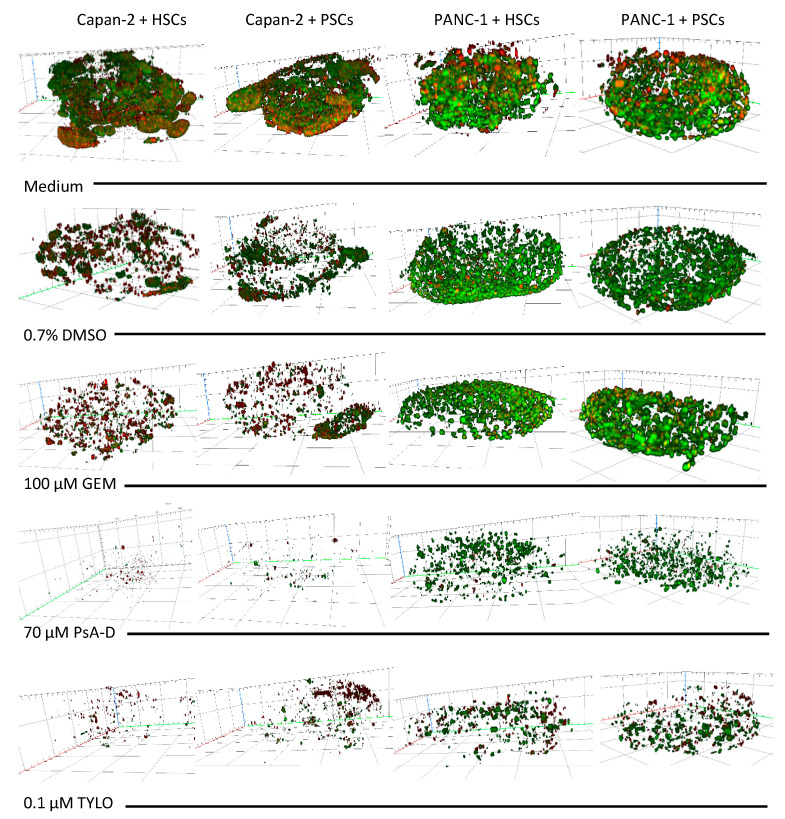
Three-dimensional rendering of the spheroids to examine the cell distribution following the exposure to test agents. Spheroids with 500 cancer cells (Capan-2 or PANC-1) and 1000 hepatic stellate cells (HSCs) or pancreatic stellate cells (PSCs) in 50 μL of 0.1 mg/mL human type I collagen were grown for 4 days and then treated with 100 μM gemcitabine (GEM), 70 μM pseudopterosin A-D (PsA-D), 0.1 μM *O*-methyltylophorinidine (TYLO), or 0.7% DMSO as a solvent control and compared to the media addition. After 3 additional days, the spheroids were fixed in paraformaldehyde (PFA) and stained for p53 (green) and Ki-67 (red). Confocal laser scanning microscopy was performed using a 10× objective lens, with image analysis and 3D rendering performed using Zen 2.3 (blue edition). Images are representative of the two independent experiments with three spheroids in each experiment. Scale bars of X, Y, and Z axes: 100 μm.

Spheroids containing cancer cells and stellate cells in human type I collagen matrices were grown for 4 days and then treated for 3 days with 100 μM GEM, 70 μM PsA-D, 0.1 μM TYLO, or 0.7% DMSO as a solvent control. Immunofluorescence assessment of Ki-67 (red) and p53 (green) of medium-treated spheroids showed a fairly consistent overall distribution of cells in Capan-2-containing spheroids that was somewhat less organized than spheroids prepared with the PANC-1 cells, with these differences being observed in all the studies performed in this format (Figure 7). While not observed in initial screening studies (data not shown), cell density in Capan-2 containing spheroids treated with 0.7% DMSO was diminished relative to medium-treated control spheroids, but such a difference between the PANC-1-containing spheroid treated with medium or 0.7% DMSO was not as striking (Figure 7). Most noticeably, and consistent with the cytotoxicity evaluation in 3D (Table 2) and the results using Airyscan imaging cells near the edge of intact spheroids (Figure 7), PsA-D and TYLO appeared to be more effective than GEM at the concentrations tested in this 3D model (Figure 7).

## 4. Discussion

While extensive progress has previously been made in treating a number of cancer entities, current chemotherapeutics have failed to provide durable benefit for PDAC patients. Since PDAC tumors are typically poorly vascularized and desmoplastic, it is possible that the lack of clinical benefit following the administration of the currently approved chemotherapeutics could be due to insufficient penetration and/or reduced cancer cell susceptibility. We addressed some aspects of these potential concerns with an in vitro model that can be used to screen drug candidates against either Capan-2 or PANC-1 PDAC cells sequestered within 3D spheroids composed of human type I collagen matrix and primary stellate cells isolated from resected human pancreatic (primary tumor) or hepatic (metastatic) PDAC lesions. Stellate cells are the most prevalent cell type in the PDAC stroma, making up about 50% of the cells present and being a major source of stromal components [3]. As gemcitabine (GEM) represents a first-line therapy for PDAC [20], we examined this agent; while somewhat more effective in Capan-2-containing 3D spheroids than PANC-1-containing 3D spheroids, GEM was clearly not as effective as expected from its observed actions in 2D cell cultures with these same PDAC cells alone. Thus, it appears that GEM, a prodrug that integrates unspecifically into DNA during synthesis to produce an irreparable error that leads to cell death [44], becomes much less effective in this non-vascularized environment containing stellate cells and type I collagen. Although in our study, we were trying to model tumor-relevant circumstances by implementing patient-derived stellate cells, which is not reflected by a 3D PDAC monoculture, it has been previously described that gemcitabine even loses efficacy from 2D to 3D PDAC monoculture, which is presumably controlled by cell–cell contacts and cell tension regulated via the Hippo pathway [45]. These findings indicate that the significant efficacy loss for GEM in 3D cannot be simply explained by the insensitivity of stellate cells against this unspecific S-phase inhibitor. Although the intercellular communication between PDAC and the activated stellate cells in the tumor microenvironment needs further elucidation, our data revealed that classical 2D screening assays are not appropriate to identify novel and efficacious drugs against PDAC.

For comparison to GEM, we performed similar studies using two natural product classes, PsA-D and TYLO; our findings showed that these agents were more effective in the in vitro 3D PDAC co-culture model. The rationale for testing the two natural products was based upon the presumed mechanism of action to display anti-inflammatory activity in the TME of triple negative breast cancer [16]. The glucocorticoid receptor alpha (GRα) appears to be overexpressed in pancreatic cancers [46,47], making it a potential target for chemotherapeutic intervention for PDAC. PsA-D has been shown to block GRα and NF-κB signaling in breast cancer cell lines grown in 2D and 3D formats [16]. While there is an association between GRα activation and NF-κB signaling [48], NF-κB signaling alone has been proposed to play a critical role in PDAC development and progression [49]. Similarly, the TYLO analogue DCB-3503 has been shown to inhibit pancreatic cancer cell growth by reducing cell cycle regulatory proteins and inhibiting NF-κB signaling [18]. Since NF-κB contributes to gemcitabine resistance in pancreatic cancer cell lines in a mechanism regulated by the mucin MUC4 [50], our findings support the possibility that the additional complexity in these 3D spheroids, relative to traditional 2D cell culture methods, may be fruitful for screening novel chemotherapeutic intervention strategies for PDAC where extracellular matrix components were incorporated.

A number of 3D models have been described previously, including reconstituted tumor organoids or genetically engineered mouse models, which incorporate various PDAC tumor-associated cells such as endothelial cells, macrophage, and infiltrating lymphocytes [9,51]. Most of these models require extensive and complex protocols that would dramatically reduce throughput rates for screening potential chemotherapeutics. The in vitro 3D spheroid system described herein was designed to provide a relatively simple system for rapidly screening potential chemotherapeutics in a model that incorporated stellate cell–cancer cell interactions and cell–matrix interactions that would occur in human PDAC tumors. In addition, the in vitro 3D spheroid model described herein may provide a tractable tool to examine the properties of potential chemotherapeutics that might limit their use in treating PDAC. These 3D spheroids could, for example, be useful in examining the extent of penetration of a chemotherapeutic agent through the collagen-dominated desmoplastic environment of PDAC tumors. This model might also be useful to assess chemical transformations/deactivations of potential chemotherapeutics occurring in the presence of stellate cells, not observed with PDAC cells alone. Furthermore, these 3D spheroids could be used to better understand the potential role of stellate cells and the desmoplastic condition of the PDAC tumors in the suppression of the growth properties of cancer cells within this environment that limits the effectiveness of chemotherapeutics designed to target rapid cell growth properties. The use of this model for such tractable studies will require improvements in detection methods and protocols.

While no in vitro model can perfectly duplicate the in situ setting in pancreatic cancer patients, this current model has hallmarks of early stage PDAC tumors that develop in patients and may provide a system sufficient for 3D screening for potential chemotherapeutics that might be more effective in the complex setting of a desmoplastic environment. The differences in efficacy observed for GEM compared to PsA-D and TYLO in this 3D spheroid model were unexpected. From the data presented, we concluded that the approach presented might not only provide a relatively fast method to screen potential chemotherapeutics for PDAC but also may provide a tractable system to examine how agents, such as GEM, might lose their expected effectiveness in the setting of PDAC tumors [52].

## Figures and Tables

**Table 1:** List of antibodies, their clonal specificities, manufacturer`s information and dilution factors used.

Antibody	Species Clonality	Manufacturer	Cat. #	Dilution
p53	Polyclonal Goat IgG	R&D systems	AF1355	1:100
p53 (DO-1)	Monoclonal Mouse IgG2a	Santa Cruz biotechnology	sc-126	1:100
αSMA	Rabbit polyclonal	Abcam	ab5694	1:100
Ki67(H-300)	Rabbit polyclonal	Santa Cruz biotechnology	SC-15402	1:100
Donkey anti-goat IgG secondary antibody, Alexa Fluo 488	Donkey polyclonal	Invitrogen/ThermoFisher	A-11055	1:200
Donkey anti-rabbit IgG secondary antibody, Alexa Fluo 546	Donkey polyclonal	Invitrogen/ThermoFisher	A10040	1:200
Chicken anti-mouse IgG secondary antibody, Alexa Fluor 647	Donkey polyclonal	Invitrogen/ThermoFisher	A-21463	1:200
Rabbit IgG Isotype Control (3 mg/mL)	Rabbit	Invitrogen	10500C	1:1000
Goat IgG (5 mg/mL)	Goat	Invitrogen	02-6202	1:500
Mouse IgG (0.4 mg/mL)	Mouse	Santa Cruz biotechnology	sc-2025	1:200

**Table 2:** IC_50_ values of cell death induced by *O*-methyltylophorinidine, pseudopterosin A-D, and gemcitabine treatment of PDAC cell lines Panc-1 and Capan-2 in 2D cell culture *.

	Gemcitabine (µM)	*O*-Methyltylophorinidine (nM)	Pseudopterosin (µM)
**PANC-1**	1.99 (±0.13)	4.6 (±0.47)	34.02 (±1.35)
**Capan-2**	0.41 (±0.06)	5.58 (±1.74)	33.94 (±1.02)

* CyQuant NF Cell Proliferation Assay (ThermoFisher) was used to determine cell viability.

**Table 3:** IC_50_ values of cell death induced by *O*-methyltylophorinidine, pseudopterosin A-D, and gemcitabine treatment of 3D co-culture spheroids composed of the PDAC cell line Panc-1 and the stellate cells PCS or HCS, respectively *.

	Gemcitabine (µM)	*O*-Methyltylophorinidine (nM)	Pseudopterosin (µM)
**PANC-1 + PSC**	>100	3.57 (±1.30)	54.42 (±12.79)
**PANC-1 + HSC**	>100	6.39 (±2.28)	51.75 (±0.60)

* Three-dimensional CellTiter Viability Assay (Promega) was used to determine cell viability.

## Supplementary Materials

The following are available online at https://www.mdpi.com/2306-5354/7/2/57/s1, Figure S1: 3D-rendering of optically-cleared spheroids using confocal laser scanning microscopy. At day 4 of culture, spheroids formed from 500 cancer cells (PANC-1 or Capan-2) and 1,000 hepatic stellate cells (HSCs) or pancreatic stellate cells (PSCs) in 50 μL of 0.1 mg/mL type I collagen were fixed and stained for αSMA (red) and p53 (green), with DAPI (blue) being used to demonstrate nuclei. Yellow arrows exemplify stellate cells. Images are representative from at least three independent experiments with at least three spheroids in each experiment. Scale bar: 100 μm; Figure S2: Comparison of microscopic images obtained from paraffin-embedded sections and optical sections. 3D co-cultures PDAC spheroids prepared from 500 Capan-2 cells and 1,000 hepatic stellate cells in 50 μL of 0.1 mg/mL type I collagen were grown for 7 days. Following fixation, paraffin-imbedding, and sectioning of some spheroids, all were stained for p53 (green), Ki67 (red), and nuclei with DAPI (blue). Immunofluorescence images were obtained using same settings on the confocal laser scanning microscope. Scale bar: 20 μm. White arrows identify an example of a cell expressing Ki67.
