# Supplementary material for: Pseudopterosin and O-Methyltylophorinidine Suppress Cell Growth in a 3D Spheroid Co-Culture Model of Pancreatic Ductal Adenocarcinoma
Source: Bioengineering (Basel). 2020 Jun 14;7(2):57. doi: 10.3390/bioengineering7020057 (PMC7357459; doi:10.3390/bioengineering7020057)
Supplement: Supplementary file 1 [file bioengineering-07-00057-s001.pdf]

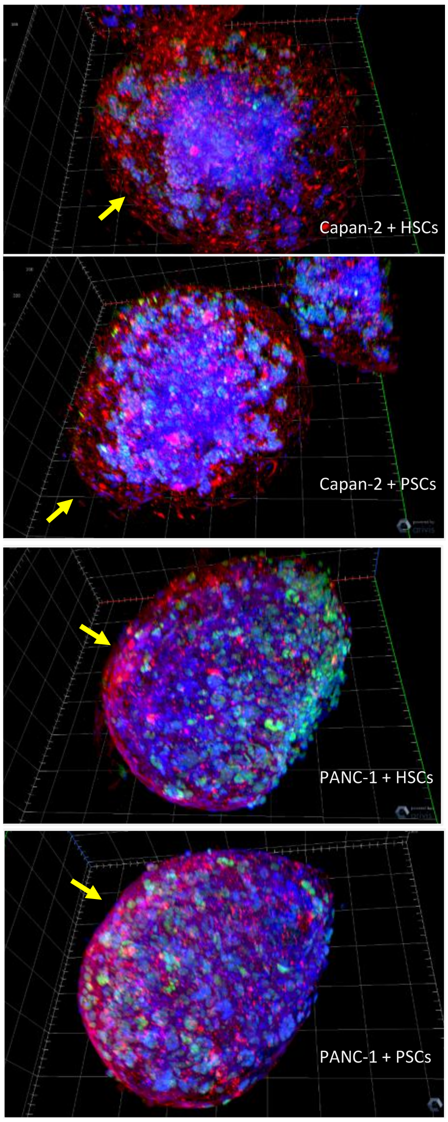

Supplementary Figure 1. 3D-rendering of optically-cleared spheroids using confocal laser scanning microscopy. At day 4 of culture, spheroids formed from 500 cancer cells (PANC-1 or Capan-2) and 1,000 hepatic stellate cells (HSCs) or pancreatic stellate cells (PSCs) in 50 μL of 0.1 mg/mL type I collagen were fixed and stained for αSMA (red) and p53 (green), with DAPI (blue) being used to demonstrate nuclei. Yellow arrows exemplify stellate cells. Images are representative from at least three independent experiments with at least three spheroids in each experiment. Scale bar: 100 μm.

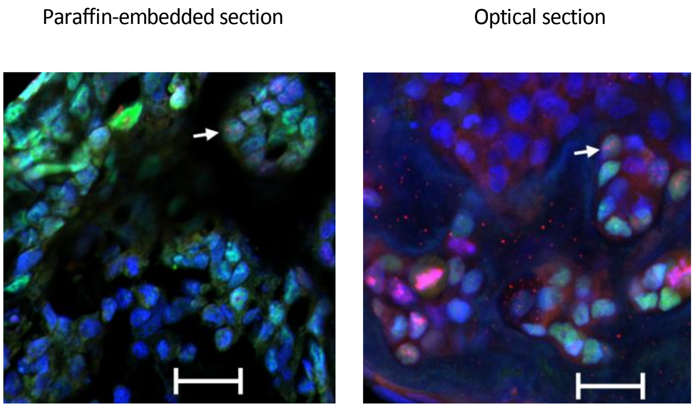

Supplementary Figure 2. Comparison of microscopic images obtained from paraffin-embedded sections and optical sections. 3D co-cultures PDAC spheroids prepared from 500 Capan-2 cells and 1,000 hepatic stellate cells in 50 µL of 0.1 mg/mL type I collagen were grown for 7 days. Following fixation, paraffin-imbedding, and sectioning of some spheroids, all were stained for p53 (green), Ki67 (red), and nuclei with DAPI (blue). Immunofluorescence images were obtained using same settings on the confocal laser scanning microscope. Scale bar: 20 µm. White arrows identify an example of a cell expressing Ki67.
